# Corticosteroid treatment for post-COVID-19 persistent interstitial lung disease: a randomized clinical trial

**DOI:** 10.1016/j.bjid.2026.105892

**Published:** 2026-07-09

**Authors:** Rosângela Villela Garcia, Marcel Koenigkam-Santos, Lucas Botelho de Azevedo, Danilo Tadao Wada, Ada Clarice Gastaldi, Elcio Oliveira Vianna

**Affiliations:** a Universidade de São Paulo, Faculdade de Medicina de Ribeirão Preto, Department of Medicine, Ribeirão Preto, SP, Brazil; b Universidade de São Paulo, Faculdade de Medicina de Ribeirão Preto, Department of Medical Imaging, Hematology, and Clinical Oncology, Ribeirão Preto, SP, Brazil; c Universidade de São Paulo, Faculdade de Medicina de Ribeirão Preto, Department of Health Sciences, Ribeirão Preto, SP, Brazil

**Keywords:** SARS-CoV-2, COVID-19, Post-COVID syndrome, Treatment, Interstitial lung disease, Pulmonary function test, High resolution chest computed tomography

## Abstract

This study aimed to evaluate the efficacy of prednisolone in the treatment of persistent interstitial lung disease following severe SARS-CoV-2 pneumonia. A single-center, randomized, double-blind, placebo-controlled clinical trial was conducted. All participants presented residual pulmonary opacities on high-resolution chest computed tomography 4-months after COVID-19 diagnosis. Patients received either prednisolone (1 mg/kg/day for 1-month followed by gradual dose reduction) or placebo and were reassessed after 3- and 6-months of follow-up. Evaluations included clinical assessment; measurement of erythrocyte sedimentation rate, C-reactive protein, and D-dimer levels; pulmonary function tests; 6-Minute Walk Test (6MWT); Modified Medical Research Council (MRC) dyspnea scale; Short Form-36 (SF-36) quality of life questionnaire; and Post-COVID-19 Functional Status (PCFS) Scale. Statistical analyses were performed using analysis of variance (ANOVA) and log-binomial regression models. The placebo group (n = 31) comprised 60% male participants with a median age of 51-years, whereas the prednisolone group (n = 35) included 52% male participants with a median age of 53-years. Baseline comorbidities and treatments prior to randomization were comparable between groups. During follow-up, forced vital capacity (% predicted) increased from 78% to 89% in the placebo group and from 83% to 91% in the prednisolone group. Significant improvements were also observed in forced expiratory volume in one second, diffusion lung capacity, and maximal expiratory pressure in both groups. No significant differences were identified between groups regarding MRC dyspnea scores, SF-36 domains, or PCFS scale at baseline or at the final evaluation. Functional capacity scores in the SF-36 increased from 55 to 71 in the placebo group and from 57 to 69 in the prednisolone group. Walking distance increased significantly only in the prednisolone group. Tomographic variables demonstrated similar progression patterns in both groups throughout follow-up. In conclusion, systemic corticosteroid therapy with prednisolone did not provide additional benefits in patients with post-COVID interstitial lung disease. Both placebo and prednisolone groups exhibited comparable functional improvements over the six-month follow-up period.

## Introduction

Severe Acute Respiratory Syndrome Coronavirus-2 is the most serious complication of COVID-19 infection and contributes to significant morbidity and mortality.^1^ The most common post-COVID-19 respiratory illness is interstitial lung disease. Previous studies have demonstrated that greater severity during the acute phase of COVID-19 is associated with a higher risk of developing persistent pulmonary interstitial abnormalities.^2^, ^3^, ^4^, ^5^

Persistent respiratory symptoms following COVID-19 infection commonly include dyspnea, fatigue, myalgia, and chronic cough. Patients who continue to experience respiratory symptoms for more than 3-months after hospital discharge are more likely to present post-COVID interstitial lung disease. Corticosteroids are widely used in the management of severe viral respiratory infections and are currently recommended in the treatment of severe acute COVID-19.^6^ However, the optimal therapeutic strategy for persistent pulmonary interstitial abnormalities after COVID-19 has not yet been clearly established.^4^^,^^7^, ^8^, ^9^

Organizing pneumonia represents the most frequent radiological pattern observed in post-COVID persistent ILD and may progress to fibrotic remodeling. Because corticosteroids are effective in the treatment of organizing pneumonia, they may also represent a potential therapeutic option for persistent post-COVID interstitial lung disease after hospital discharge.^4^^,^^10^, ^11^, ^12^, ^13^, ^14^ Indeed, Oliveira-Filho et al. were the first authors to address the question of whether an approach with a high dose of corticosteroids would be beneficial for patients with organizing pneumonia secondary to COVID-19. They reported a series of three cases with organizing pneumonia that responded dramatically to corticosteroids.^11^ The potential benefits of corticosteroid therapy in post-COVID lung disease remain uncertain in the current literature. Consequently, this topic continues to represent an important unresolved research question, underscoring the relevance of the present study.

### *Aims*

Based on the hypothesis that corticosteroid therapy could improve outcomes in patients with persistent post-COVID interstitial lung disease, the present study aimed to evaluate the effects of prednisolone treatment in individuals with persistent ILD following severe SARS-CoV-2 pneumonia.

## Methods

### *Study design*

This was a single-center, randomized, double-blind, placebo-controlled clinical trial involving patients previously diagnosed with SARS-CoV-2 pneumonitis who had been admitted to the Hospital das Clínicas de Ribeirão Preto, University of São Paulo, Brazil. Participants with persistent post-COVID interstitial lung disease were enrolled and randomized 4-months after COVID-19 diagnosis. Patients were subsequently followed for 6-months after randomization. Follow-up visits were conducted 3-months (V1) and 6-months (V2) after randomization.

The study was registered at ClinicalTrials.gov under registration number NCT07380152.^15^

### *Patients*

The study included adults older than 18-years with a history of hospitalization for SARS-CoV-2 pneumonia and persistent respiratory symptoms after recovery. Persistent symptoms included chronic cough, dyspnea, and fatigue. Eligibility criteria required tomographic evidence of interstitial lung disease 4-months after the initial diagnosis, characterized by ground-glass opacities, reticular opacities with peripheral predominance, parenchymal bands, or fibrotic stripes.

All participants presented at least 25% pulmonary involvement on High-Resolution Computed Tomography (HRCT). Chest CT scans were performed without intravenous contrast by using multidetector scanners according to HRCT protocols recommended by the American Thoracic Society/European Respiratory Society and the Fleischner Society for the evaluation of interstitial lung and airway diseases.^16^ To ensure consistency in image interpretation, all imaging examinations considered for study inclusion were initially evaluated by a single radiologist. Subsequently, a definitive assessment was established through an independent review conducted by two additional radiologists.

The study protocol was approved by the Institutional Review Board, and written informed consent was obtained from all participants prior to enrollment.

Exclusion criteria included alternative causes for hospital admission, such as bacterial pneumonia, pulmonary embolism, acute myocardial infarction, or heart failure during hospitalization, as well as uncontrolled insulin-dependent diabetes mellitus, glaucoma, uncontrolled psychiatric disorders, active cancer under treatment, and neurological impairment preventing the completion of study procedures.

### *Clinical assessment*

Patients underwent structured assessment through a multidisciplinary team. This assessment included medical evaluation, D-dimer, C-Reactive Protein (CRP), Erythrocyte Sedimentation Rate (ESR), Electrocardiogram (ECG), 6-Minute Walking Test (6MWT), Short Form-36 Questionnaire score (SF-36), Current Medical Research Council (MRC) dyspnea score, Post-COVID-19 Functional Status (PCFS) scale, lung function tests with Forced Vital Capacity (FVC), Forced Expiratory Volume in one second (FEV1), Total Lung Capacity (TLC), Diffusing capacity of the Lungs for Carbon monoxide (DLCO), Maximal Inspiratory Pressure (MIP), maximal expiratory pressure (MEP), and HRCT imaging. The primary outcome was FVC, expressed in Liters (L).

Following baseline assessment, participants-initiated treatment and underwent reevaluation after 3- and 6-months.

### *Treatment*

Participants were randomly assigned to receive either placebo or corticosteroid therapy. Patients in the treatment group received prednisolone at a dose of 0.5 mg/kg/day for 30 days, followed by progressive dose tapering: half-dose for seven days, one-quarter dose for seven days, and one-eighth dose for seven days before discontinuation. The placebo group received an identical number of tablets administered over the same period.

### *Randomization*

The treatment capsules (placebo and prednisolone) were prepared by a compounding pharmacy. A study team member (EOV), who was not involved in patient recruitment and had no access to patient data or study procedures, was responsible for organizing and randomizing the treatment vials. Randomization was performed using a numerical sequence from 1 to 100, with numbers randomly allocated to the two groups (placebo and prednisolone). Treatments were identified exclusively by these numerical codes. Allocation concealment was maintained for both the participants and the research team directly involved in medical record screening, recruitment, and study procedures.

### *Statistical analysis*

As this is a relatively rare condition and the present study represents an early investigation of corticosteroid therapy for post-COVID disease, all patients from our institution who met the inclusion criteria were enrolled. Therefore, no prospective sample size calculation was performed. Interpretation of the findings was based primarily on effect estimates and their corresponding 95% Confidence Intervals, allowing for the assessment of the magnitude, direction, and precision of the observed effects.

Longitudinal analyses between groups and time points were conducted using all participants with valid information available at each visit through repeated-measures models. Analyses of changes over time (delta between visits) included only participants with data available at both compared time points. Accordingly, the analytical strategy approximated a modified intention-to-treat approach, considering all participants with at least one post-baseline assessment available.

No imputation of missing data was performed. Analyses were conducted using all valid observations available at each visit through repeated-measures models. Model assumptions were assessed by graphical inspection of residuals and formal tests of normality; transformations or alternative statistical models were applied when necessary. Although multiple outcomes were evaluated, analyses were guided by predefined clinical hypotheses, some of which were exploratory in nature. Consequently, formal global adjustments for multiple comparisons were not performed, as such procedures could result in excessively conservative corrections in a study with a relatively limited sample size. Therefore, results were interpreted in conjunction with effect estimates, confidence intervals, and the clinical relevance of the findings. In addition, baseline demographic and clinical characteristics were comparable between groups, making further adjustment for potential confounders unnecessary.

Although no formal sensitivity analysis was performed, absolute changes (delta) between follow-up visits were also analyzed using only participants with valid data at the respective time points, serving as a complementary assessment of result consistency.

Repeated-measures ANOVA models were used to evaluate continuous variables, including FEV1, FVC, TLC, DLCO, MIP, MEP, 6MWT distance, ESR, CRP, and D-dimer levels.

SF-36 scores were analyzed using a repeated-measures negative binomial regression model with a logarithmic link function, since these outcomes may be considered discrete quantitative variables. This approach was chosen because the assumptions of normality and homoscedasticity of residuals required for ANOVA were not met. Relative increases or reductions in mean values were calculated from the model estimates using the expression: AR(β) = [exp(β) − 1] × 100%.

The mMRC dyspnea scale and PCFS scale, both ordinal categorical variables, were analyzed using ordinal multinomial regression model, which estimates the odds ratio for higher scores, corresponding to worse clinical status.

The dichotomized PCFS analysis was performed using a log-binomial regression model, estimating the prevalence ratio for grades 3 or 4 according to study group and follow-up time point.

## Results

During the initial screening of 2388 patients, the medical records of all individuals hospitalized with an ICD code related to COVID-19 were reviewed. Patients who did not meet criteria for severe COVID-19 (ICD-10 codes U07.1 and B34.2) or who fulfilled any of the predefined exclusion criteria were excluded. Subsequently, 322 eligible patients were contacted by telephone or messaging application (WhatsApp).

**Fig. 1:**
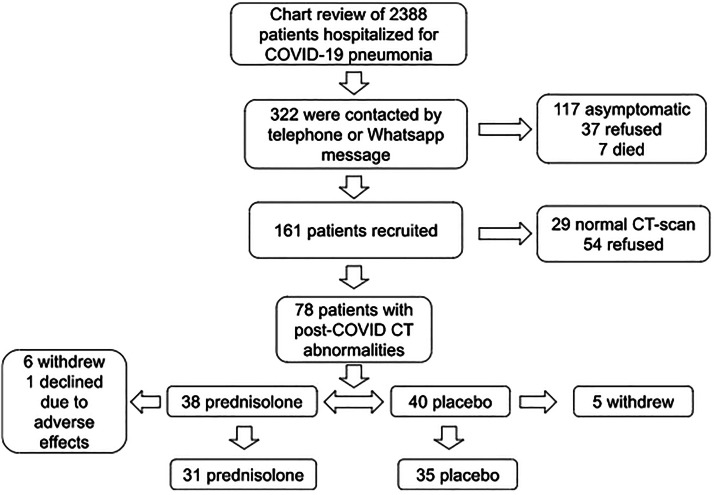
Flowchart illustrating the recruitment process and selection of patients included in the study.

At the screening stage, 117 patients reported complete resolution of symptoms, 37 declined participations, and 7 had died. As a result, 161 patients presenting persistent symptoms were recruited for clinical evaluation (Fig. 1). During the initial visit, 54 participants refused enrollment in the placebo-controlled trial, and 29 failed to meet the inclusion criteria. Consequently, a total of 78 patients underwent randomization (Fig. 1).

One patient in the prednisolone group discontinued treatment because of corticosteroid-related adverse effects, including insomnia, irritability, and agitation. An additional 11 patients withdrew from the study during follow-up (Fig. 1). Consequently, the final analysis included 31 patients in the prednisolone group and 35 patients in the placebo group.

The most common adverse effects were actively monitored throughout the period. Significant weight gain was observed in both groups, predominantly among patients who were already obese and had experienced weight loss during prolonged hospitalization. Glycemic control was not systematically assessed, except in patients with previously diagnosed diabetes mellitus, for whom medication adjustments were performed as needed during treatment. No participants reported proximal muscle weakness. Symptoms of anxiety and insomnia were among the most frequently reported adverse effects; however, several patients had already experienced these symptoms persistently following hospitalization. These adverse effects led to treatment discontinuation in only one participant.

All participants had been diagnosed with severe COVID-19 pneumonia, and the therapeutic interventions administered for respiratory failure during hospitalization are summarized in Table 1. Study inclusion occurred at a mean interval of 118.2 ± 19.9 days after the diagnosis of COVID-19 (mean ± standard deviation).

**Table 1:** Patients characteristics and previous COVID-19 treatment by group.

	**Placebo Group (n = 35)**	**Prednisolone Group (n = 31)**	**p-value**
**Demographics**			
Age (median, IQR)	51 (32 ‒ 69)	53 (28 ‒ 78)	0.36
Female sex, n (%)	14 (40.0%)	15 (48.4%)	0.62
White or Asian, n (%)	25 (71.5%)	16 (51.6%)	
Black, n (%)	10 (28.5%)	24 (77.5%)	0.78
**Comorbidities, n (%)**			
Body mass index < 30	12 (34.3%)	11 (35.5%)	
Body mass index ≥ 30	23 (65.7%)	20 (64.5%)	0.78
Current or former smoker	5 (14.3%)	4 (12.9%)	0.99
Hypertension	13 (37.1%)	12 (38.7%)	0.99
Diabetes mellitus	5 (14.3%)	5 (14.3%)	0.99
Dyslipidemia	5 (14.3%)	4 (12.9%)	0.99
**COVID-19 Treatment during hospitalization previously to recruitment**			
Respiratory failure treatment, n (%)			
Noninvasive O_2_	6 (17.1%)	6 (19.4%)	0.99
CPAP or HFNC	11 (31.4%)	10 (32.3%)	
IMV	17 (48.6%)	15 (48.4%)	
Intubation period (days)	16.7 (5 ‒ 42)	21.3 (8 ‒ 44)	0.75
Corticosteroids use (days)	17.2 ± 12.2	18.6 ± 14.4	0.78

IQR, Interquartile Range; O_2_, Oxygen therapy; CPAP, Continuous Positive Airway pressure; HFNC, High Flow Nasal Cannula; IMV, Invasive Mechanical Ventilation.

The placebo and prednisolone groups were comparable with respect to demographic characteristics, including sex, ethnicity, and age. Additionally, no significant differences were observed between groups regarding comorbidities, such as obesity, smoking status, hypertension, diabetes mellitus, and dyslipidemia, nor in relation to disease severity and treatments received during hospitalization (Table 1).

No significant differences were observed between the placebo and prednisolone groups regarding inflammatory markers either at baseline or during follow-up visits (V1 and V2). Baseline ESR values were elevated relative to the reference range in both groups, with mean values of 25.4 mm/1st hour (SD = 21.97) in the placebo group and 31.58 mm/1st hour (SD = 30.34) in the prednisolone group and progressively decreased at V1 to 14.03 mm/1st hour (SD = 12.15) and 21.1 mm/1st hour (SD = 23.43), respectively, and further declined at V2 to 9.66 mm/1st hour (SD = 6.33) and 15.57 mm/1st hour (SD = 14.10), respectively. These reductions were statistically significant over time (p < 0.05).

C-Reactive Protein (CRP) concentrations remained within the normal reference range (< 1.0 mg/dL) throughout the study period and did not differ significantly between groups. At baseline, mean CRP-values were 0.70 mg/dL (SD = 0.70) in the placebo group and 1.06 mg/dL (SD = 2.83) in the prednisolone group, at V1, corresponding values were 0.77 mg/dL (SD = 0.77) and 0.77 mg/dL (SD = 0.83), respectively, whereas at V2, mean values were 0.74 mg/dL (SD = 0.71) and 0.79 mg/dL (SD = 0.81), respectively.

D-dimer levels remained stable throughout follow-up, with no significant differences observed between groups.

All spirometric parameters demonstrated improvement from baseline to follow-up visits in both groups, without significant intergroup differences (Table 2). Baseline values for TLC, DLCO, MIP, and MEP were comparable between groups. Notably, DLCO and MEP values showed significant improvement from baseline to V2 in both treatment groups (Table 2).

**Table 2:** Pulmonary function data at baseline, V1, and V2 by group.

	**Placebo Group (n = 29)**			**Prednisolone Group (n = 28)**		
**Variables**	**Baseline**	**V1**	**V2**	**Baseline**	**V1**	**V2**
FEV1 (L)	2.63 ± 0.65	2.83 ± 0.61^a^	2.75 ± 0.63^a^	2.67 ± 0.89	2.86 ± 0.97^a^	2.85 ± 0.78^a^
FEV1 (%)	79.7 ± 14.4	85.9 ± 13.3^a^	88.2 ± 14.1^a^	84.5 ± 18.7	89.8 ± 18.9^a^	92.3 ± 13.7^a^
FVC (L)	3.20 ± 0.85	3.58 ± 0.85^a^	3.47 ± 0.85^a^	3.25 ± 1.03	3.49 ± 1.13^a^	3.49 ± 0.96^a^
FVC (%)	78.1 ± 14.9	86.9 ± 14.1^a^	88.8 ± 16.2^a^	82.7 ± 16.1	88.2 ± 16.4^a^	90.7 ± 13.2^a^
TLC (L)	4.49 ± 1.18	4.82 ± 1.19	5.12 ± 1.28	5.18 ± 2.85	5.43 ± 3.17	4.78 ± 1.45
TLC (%)	73.9 ± 13.2	80.4 ± 10.2	85.8 ± 8.5	91.6 ± 36.4	94.7 ± 40.6	86.6 ± 13.8
DLCO (%)	73.8 ± 15.1	80.1 ± 14.4^a^	88.7 ± 14.6^a^	72.8 ± 13.1	73.9 ± 12.9	80.9 ± 15.3^a^
MIP (cmH_2_O)	66.8 ± 30.9	77.7 ± 36.2	86.0 ± 36.7	79.2 ± 25.4	81.2 ± 26.7	87.5 ± 25.6
MEP (cmH_2_O)	81.6 ± 26.0	86.4 ± 25.1	112.8 ± 30.0^a^	86.0 ± 19.6	85.5 ± 19.5	107.9 ± 30.5^a^

FEV1 (L), Forced Expiratory Volume in one second expressed as absolute value (liter); (%), Expressed as percentage of predicted value; FVC, Forced Vital Capacity; TLC, Total Lung Capacity; DLCO, Diffusion Lung Capacity; MIP, Maximal Inspiratory Pressure; MEP, Maximal Expiratory Pressure; V1, First follow up visit at 3-months; V2, Second follow up visit at 6-months.

Data are shown as mean ± SD.

a p-value < 0.05 for the comparison with baseline.

No significant differences were observed in Medical Research Council (MRC) scale scores between the placebo and prednisolone groups at any study visit (Table 3).

**Table 3:** Functional assessment at baseline, V1, and V2 by group.

	**Placebo Group (n = 35)**			**Prednisolone Group (n = 30)**		
**Variables**	**Baseline**	**V1**	**V2**	**Baseline**	**V1**	**V2**
**Dyspnea MRC ≥2**	16 (46%)	15 (43%)	7 (20%)	13 (43%)	11 (37%)	10 (33%)
**SF-36 questionnaire**						
Functional capacity	54.7 ± 24.3	69.9 ± 23.4^a^	70.9 ± 24.3^a^	57.3 ± 32.1	66.2 ± 27.6	68.5 ± 30.2^a^
Functional limitation	26.4 ± 37.8	48.5 ± 45.0^a^	43.1 ± 44.8	30.0 ± 39.1	44.0 ± 41.0	44.4 ± 42.4
Pain	51.1 ± 27.9	62.9 ± 28.0^a^	56.1 ± 29.9	54.2 ± 31.9	61.7 ± 25.1	59.2 ± 27.6
General state	32.7 ± 11.7	34.1 ± 11.6	35.0 ± 12.3	35.9 ± 7.9	35.5 ± 10.3	37.3 ± 9.3
Vitality	51.6 ± 24.8	60.3 ± 25.3^a^	56.7 ± 26.4	57.2 ± 22.8	60.2 ± 18.9	59.8 ± 22.6
Social aspects	68.6 ± 27.9	70.8 ± 29.9	72.9 ± 31.2	67.1 ± 33.2	75.4 ± 23.5	70.4 ± 28.4
Emotion limitation	50.5 ± 43.1	71.7 ± 39.2^a^	54.0 ± 44.0	65.6 ± 40.6	59.8 ± 43.1	68.0 ± 35.2
Mental health	77.0 ± 19.5	78.9 ± 20.7	79.0 ± 20.4	81.7 ± 14.1	81.2 ± 9.0	80.6 ± 13.9
**PCFS Scale**						
Score ≤ 2	22 (63%)	29 (83%)	27 (77%)	18 (60%)	21 (70%)	21 (70%)
Score 3 or 4	13 (37%)	6 (17%)	8 (23%)	12 (40%)	9 (33%)	9 (33%)
**6MWT**						
Distance in meters	470 ± 85	480 ± 75	486 ± 80	452 ± 153	459 ± 131	507 ± 99^a^

MRC, Dyspnea Score by Medical Research Council; PCFS Scale, Post-COVID-19 Functional Status Scale; 6MWT, Six-minute walking test; V1, First follow up visit at 3-months; V2, Second follow up visit at 6-months.

Data are shown as n (%) or mean (SD).

a p-value < 0.05 for the comparison with baseline.

Analysis of the SF-36 questionnaire demonstrated no significant differences between groups in the overall score derived from the eight domains evaluated. Functional capacity improved significantly in both groups from baseline to V2. In the placebo group, significant improvements from baseline to V1 were observed in the domains of functional limitation, pain, vitality, and emotional limitation (Table 3).

Assessment using the Post-COVID-19 Functional Status (PCFS) scale demonstrated transient improvement in both groups; however, these effects were not sustained over time. No significant differences between the placebo and prednisolone groups were identified at any evaluation time point.

Six-minute walk test distances are presented in Table 3. No significant differences between groups were detected during follow-up. Nevertheless, in the prednisolone group, the walking distance measured at V2 was significantly greater than the baseline value.

No significant differences in radiological findings were observed between groups or across the different evaluation time points (Table 4).

**Table 4:** High resolution chest computed tomography at baseline, V1, and V2 by group.

	**Placebo (n = 35)**			**Prednisolone (n = 31)**		
**Findings**	**Baseline**	**V1**	**V2**	**Baseline**	**V1**	**V2**
Diffuse ground glass	20 (57%)	24 (69%)	21 (60%)	19 (61%)	16 (52%)	18 (58%)
Mosaic attenuation	34 (97%)	32 (91%)	31 (89%)	29 (94%)	29 (94%)	30 (97%)
Mosaic pattern	23 (66%)	25 (71%)	19 (54%)	21 (68%)	22 (71%)	21 (68%)
Lung architectural distortion	26 (74%)	24 (69%)	23 (66%)	19 (61%)	18 (58%)	18 (58%)
Parenchymal bands	15 (43%)	12 (34%)	17 (49%)	18 (58%)	16 (52%)	18 (58%)
Traction bronchiectasis	21 (60%)	21 (60%)	19 (54%)	22 (71%)	21 (68%)	20 (65%)
Airway disease	8 (23%)	8 (23%)	12 (34%)	7 (23%)	11 (35%)	11 (35%)
Radiology improvement		20 (57%)	13 (37%)		15 (48%)	11 (35%)

V1, First follow up visit at 3-months; V2, Second follow up visit at 6-months.

Data are shown as n (%) of individuals with that radiological finding.

## Discussion

There is currently no definitive or widely accepted treatment for patients with persistent interstitial lung disease following SARS-CoV-2 infection. Corticosteroid therapy has been proposed as a potential therapeutic strategy, and this hypothesis was investigated in the present study.

The decision to evaluate patients approximately four months after the COVID-19 diagnosis was intended to include only individuals with persistent (chronic) pulmonary abnormalities while excluding those with transient post-infectious inflammatory changes. Consequently, the study aimed to assess treatment effects specifically in patients with established persistent lung disease rather than those with acute resolving disease.

In this randomized, double-blind, placebo-controlled trial, we included patients with persistent post-COVID symptoms associated with diffuse parenchymal lung abnormalities. The study evaluated whether corticosteroid therapy with prednisolone could improve respiratory function and quality of life. Baseline characteristics, including COVID-19 severity and risk factors, were similar between the placebo and prednisolone groups. The study population showed a high prevalence of male sex and obesity, and systemic arterial hypertension was the most frequent comorbidity (38%).

Pulmonary function assessment included FEV1, FVC, DLCO, TLC, MIP, and MEP. No significant differences were observed between groups at baseline, the 3-month follow-up, or the 6-month follow-up evaluations. Both groups demonstrated significant and consistent improvements over time in FEV1, FVC, DLCO, and MEP.

The erythrocyte sedimentation rate was elevated at baseline in both groups and progressively decreased during follow-up. C-reactive protein and d-dimer levels remained mildly elevated throughout the study, without significant differences between groups. Overall, oral corticosteroid therapy did not demonstrate additional benefit in reducing markers of systemic inflammation.^17^^,^^18^

In addition, no differences were found between groups regarding functional evaluation by the following questionnaires: MRC, SF-36 (sum of eight sessions), and similarly, no significant differences were observed between groups in functional assessments using the mMRC dyspnea scale, SF-36 questionnaire (total score across the eight domains), or the Post-COVID-19 Functional Status (PCFS) scale. At the first follow-up evaluation, the placebo group showed improvements in the SF-36 domains of functional limitation, pain, and vitality. Improvement in the emotional limitation domain was also observed only in the placebo group at the 3-month follow-up. These findings may be related to corticosteroid-associated adverse effects, which could have negatively influenced emotional and subjective quality-of-life outcomes in the prednisolone group.

Regarding the 6MWT, walking distance increased significantly at the 6-month evaluation compared with baseline only in the prednisolone group. Because improvements in pulmonary function were observed similarly in both groups, the increase in walking distance appears to be dissociated from pulmonary recovery. This finding suggests that improved exercise capacity may be related to corticosteroid effects on muscle function and/or exercise performance rather than direct improvement in interstitial lung disease. These results contribute to the understanding of exertional limitations in post-COVID patients.^19^^,^^20^ One possible explanation is that persistent inflammatory muscle involvement associated with long COVID may be partially reversible with anti-inflammatory therapy.

Radiological findings evolved similarly in both groups. There was a dissociation between radiological and functional recovery, with no substantial radiological improvement observed during follow-up. It is possible that radiological recovery occurs later than functional recovery. Therefore, longer follow-up periods and more detailed radiological analyses are necessary to better define the extent of radiological resolution and the presence of permanent pulmonary sequelae.

Mizera et al. (2024)[7] treated 131 patients with a mean corticosteroid treatment duration of 13.3-weeks and reported improvements in pulmonary function, radiological findings, and subjective symptoms. Another study involving 30-patients initiated corticosteroid therapy 4-weeks after hospital discharge and demonstrated significant improvements in radiological abnormalities as well as physiological and functional deficits.^12^ However, both studies were open-label and non-randomized.

Dhooria et al. evaluated corticosteroid therapy in a randomized open-label trial comparing high-dose prednisolone (40 mg/day for one week followed by tapering of 10 mg/week) with low-dose prednisolone (10 mg/day for six weeks) in patients with symptomatic post-COVID diffuse parenchymal lung abnormalities. Both regimens resulted in similar improvements in clinical, radiological, physiological, and health-related quality-of-life outcomes assessed by the SF-36 questionnaire.^4^

The present findings also contribute to the understanding of the natural history of untreated persistent post-COVID interstitial lung disease. Patients in the placebo group demonstrated progressive improvement in most evaluated variables. Similarly, a recent Brazilian study reported that six months after severe COVID-19, persistent symptoms, impaired pulmonary function, and reduced performance on the 6MWT remained present in approximately 28%, 16%, and 25% of patients, respectively.^20^ In both studies, FVC increased from approximately 80% of the predicted value at the initial evaluation to approximately 90% at the 6-month follow-up. Additional long-term studies are required to determine the extent of irreversible pulmonary damage.

The strengths of this study include the standardized evaluation protocol with chest computed tomography, which allowed accurate characterization of post-COVID interstitial lung disease and exclusion of pulmonary embolism or other pulmonary disorders. Pulmonary function assessment was comprehensive and included lung diffusion capacity, respiratory muscle pressures, and lung volumes measured by plethysmography. Functional capacity assessment was also extensive.

However, several limitations should be acknowledged. First, the corticosteroid regimen, including drug selection, dosage, timing of administration, and duration of therapy, was based on protocols commonly used for organizing pneumonia and may not represent the optimal treatment strategy for persistent post-COVID pulmonary abnormalities. Second, short- and long-term corticosteroid adverse effects were not comprehensively evaluated. In addition, spontaneous recovery observed in both groups may account for a substantial proportion of the functional improvements identified during follow-up.

In summary, corticosteroid therapy for persistent post-COVID interstitial lung disease did not demonstrate significant benefits in pulmonary function, radiological findings, or quality of life in this study, although the relatively small sample size limits definitive conclusions. A slight worsening of emotional symptoms was observed in the corticosteroid group, possibly related to treatment adverse effects. Conversely, the significant improvement in 6MWT distance observed only in the prednisolone group suggests a potential beneficial effect on post-COVID muscular dysfunction. Further randomized studies with larger sample sizes are needed to better determine the role of corticosteroid therapy in patients with persistent post-COVID interstitial lung disease.

## Conclusion

This study did not demonstrate a significant benefit of systemic corticosteroid therapy for patients with persistent post-COVID-19 symptoms and interstitial lung disease. The clinical course of this chronic condition is characterized by spontaneous, progressive recovery of pulmonary function, thereby confounding the detection of treatment effects, particularly within limited sample sizes.

## Funding

This work was supported by the São Paulo Research Foundation ‒ FAPESP, process number: 2017/21035-8, and by FAEPA.

## Data availability

The data that support the findings of this study are available from the corresponding author upon reasonable request.

## Conflicts of interest

The authors declare no conflicts of interest.
